# Obituary Notices

**Published:** 1858-11

**Authors:** 


*Obituary Notices.—Death of Doctor Billings.*—Died on the 2d September, at his residence in the town of Batavia, James Avery Billings, M. D. He was the eldest son of Perez Billings, and was born on the 4th of January, 1795, in the town of Milton, in the county of Saratoga. He studied medicine first with Dr. Billy J. Clark, of that county, and graduated at the New York University, in 1818. The late Dr. David Hossack, and the present venerable Dr. Mott, were among his professors.

He came to this county in 1818, and soon after purchased the land upon which he continued to reside until his death. This land was the first lot deeded by the Holland Land Company in the township, (T. 12, R. 1), which now embraces portions of the towns of Batavia and Stafford.

Dr. Billings was a man of sound judgment and more than ordinary ability. His practice was uniformly as extensive as the most active pursuit of it would allow. No man better understood the various conditions of a new country, or sympathized more sincerely with the privations and trials of those who were at once his neighbors, his patrons, and his friends. His unselfish exposure by night and by day, and the fatigues incident to these and to a country comparatively new, impaired a constitution otherwise strong, and of late years told with unerring aim upon his health.

He was one of the delegates from the Medical Society of Genesee County, to the National convention at Washington the past winter, and was taken down while on his return, with a paralysis, from which he but partially recovered.

He was a firm and faithful member of the Episcopal Church; and as a true and loyal friend of his country and its institutions, he helped materially to give character and support to the Democratic party, with which he was prominently identified, and often was one of its chosen leaders.

He married twice, and leaves a widow and five children, the inheritors of his good name and of a good property.

His funeral was probably the largest which ever took place in the county, and the downcast look and tearful eye were the silent but impressive monitors, indicating to all around that one greatly beloved had gone to

> "——— that undiscovered country
> From whose bourne no traveler returns."

—*Batavia Democrat.*

Prof. MAUTHNER, Director of the St. Anne's Children's Hospital, Vienna, author of numerous valuable essays, and editor of the *Journal für Kinderkrankheiten*, died of meningitis, at Vienna, in the prime of life.

JOHANNES MULLER, the distinguished Physiologist, died suddenly, while asleep, at Berlin, on the 28th of April last, in the 56th year of his age.

Dr. WILLIAM GREGORY, son of the famous James Gregory, and Professor of Chemistry in the University of Edinburgh, died on the 24th of April, after a protracted illness. He was a private pupil of Liebig, and translated several of his works; he was himself also the author of several standard treatises.

On the preceding day, Dr. ROBERT HARRISON, Professor of Anatomy in Trinity College, Dublin, and author of the well known "Dublin Dissector," died of apoplexy, in the 63d year of his age.

Sir JAMES MCGREGOR, the Director-General of the British Army Medical Staff for more than thirty-five years, recently died in England, at the unusually old age of 86.

Sir PHILIP CRAMPTON, one of Ireland's most distinguished surgeons, lately died in Dublin, at the age of 82.

Dr. JOHN SNOW, an eminent physician, well known for his researches on chloroform and other anæsthetics, and the discoverer of amylene, died of apoplexy, on the 16th of June last.

Dr. MEREI, Professor of the History of Medicine in the University of Pesth, founder of the great Children's Hospital in that city, one of the first medical men in Hungary, and an eminent literary scholar, died on the 12th of March, aged 54.—*Charleston Med. Journal.*

